# Antibacterial activity of herbal formulation against common oral pathogens

**DOI:** 10.6026/97320630019663

**Published:** 2023-05-31

**Authors:** Remmiya Mary Varghese, Subramanian Aravind Kumar, S Rajeshkumar

**Affiliations:** 1 Department of Orthodontics, Saveetha Dental College and Hospitals, Saveetha Institute of Medical and Technical Science, Saveetha University, Chennai, Tamil Nadu

**Keywords:** *Ocimum gratissimum*, *Ocimum sanctum*, tulsi, minimum inhibitory concentration, Brine shrimp lethality assay

## Abstract

The development of antibiotic resistance in microorganisms is a global challenge for the clinicians, pharmacist and research scientists leading to the development of new medicinal formulations that are effective and easily consumable. The plant yielding essential oil with chief constituent as eugenol has been identified as an important compound with strong inhibition of bacteria, and storage fungi. *Ocimum gratissimum* and *Ocimum sanctum* is an aromatic shrub occurring in warm tropical regions has been used in traditional medicine in India to cure various ailments in general and as an antimicrobial agent in particular. The aim of this present study is to assess the antimicrobial and cytoxic activity of the formulation against oral pathogens. The formulation of O. gratissimum and *O. sanctum* plant extract was prepared and filtered. Antimicrobial activity was done by agar well diffusion method, minimum inhibitory concentration assessment was determined by broth dilution method and cytotoxicity was assessed by brine shrimp lethality assay. Agar well diffusion method against *S. mutans,* *Enterococcus faecalis*, *C. albicans*, *Lactobacillus sp,* and *S. aureus* revealed no zone of inhibition but at 100µL concentration at every time interval, the study formulation showed more bacteriostatic activity than positive control and the standard used. The formulation showed very minimal cytotoxicity. The formulation of O. gratissimum and *O. sanctum* synergistically showed more antibacterial, antifungal and cytotoxic activity and more research has to be done in invivo environment.

## Background:

Herbalism has become incredibly popular worldwide during the past century. Plants continue to play a significant role in healthcare despite the significant advancements in contemporary medicine. This is because traditional medical practices, particularly those with Asian antecedents, are valued and because powerfully curative herbs from indigenous pharmacopeias have been identified. Despite being found all across the world, tropical nations have the greatest proportion of medicinal plants [2,3]. *Ocimum gratissimum L.* (African Basil - common name) is an aromatic medicinal herb. Not only among Kenyan communities but also throughout sub-Saharan Africa, it is a significant herbal medicinal plant [4]. There are an estimated 80,000 species of higher plants in Brazil alone, which presents a vast opportunity for the discovery of novel drugs. In tropical and warm temperate climates, *Ocimum gratissimum* (labiatae) is distributed widely. When nostrils are clogged, the leaves are rubbed between the palms and inhaled [5]. The plant is frequently used in traditional medicine to treat a variety of illnesses, including pneumonia, cough fever, conjunctivitis, headaches, diarrhoea, ophthalmic, skin, and eye infections. It has been observed that a number of Ocimum species and cultivars produce a variety of oils known as basilica oils [6]. Eugenol, linalol, methyl cinnamate, camphor, and thymol were among the chemical components and active substances identified in these plants [7, 8]. There have been several reports of the usage of various Ocimum species for medicinal purposes. The antibacterial qualities of this plant have also been extensively studied in relation to a few particular pathogens. For instance, it has been observed that *O.gratissimum* is effective against various kinds of bacteria and fungi [9]. Not only in Africa, has India also had its own heritage of medicinal herbs. One such herb which is used in common households for centuries as a part of medicinal and religious value is Tulsi. Hailing from the same family Lamiaceae, black tulsi's scientific name is Ocimum tenuiflorum (also known as *Ocimum sanctum*) [10]. Due to its renowned medical properties, tulsi, also known as holy basil, has been called the "Queen of plants" and the "mother medicine of nature." It has long been one of the most revered and comprehensive herbs used in Indian traditional medicine, and it has been discovered that practically every portion of the plant has therapeutic effects [11]. Tulsi is traditionally utilised in numerous ways, such as aqueous extracts from the leaves (either fresh or dried as powder) that are added to herbal teas or blended with other herbs or honey to increase its medicinal efficacy [12]. Aqueous preparations of Tulsi are traditionally used to treat a range of poisonings, stomachaches, the common cold, headaches, malaria, inflammation, and heart disease [13]. Tulsi's leaves and inflorescence are used to make oils that have been claimed to have a variety of beneficial effects, including those for expectorants, analgesics, antipyretics, and antiemetics; reducing stress and inflammation; and acting as anti-asthmatic, hypoglycemic, hepatoprotective, hypotensive, hypolipidemic, and immuno-modulatory agents [14, 15]. Hence, African basil and Indian black tulsi formulations were assessed for antimicrobial, antifungal and cytotoxic effectiveness in an in vitro setup.

## Material and Methods:

## Preparation of African Basil and Black Tulsi:

**Figure 1:**
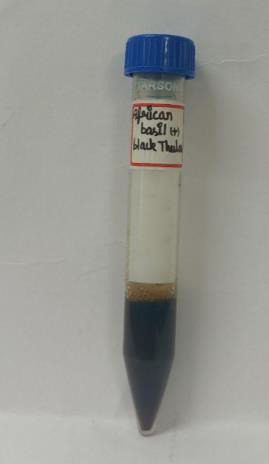
Synthesis of Africal basil and Black Tulsi formulation

The aqueous extract of African basil and black tulsi was made by boiling 2.5 g of African basil powder and 2.5 g of black tulsi powder in 100 ml of double-distilled water in a water broth at 40-60°C for 15-20 min to get 1% of the extract. After being filtered, plant extract was once more placed in the heating mantle to condense and reach a volume of 5 mL. For future research, the plant extract was then preserved in the centrifuge tube and refrigerated (Figure 1).

## Antimicrobial assay:

**Figure 2:**
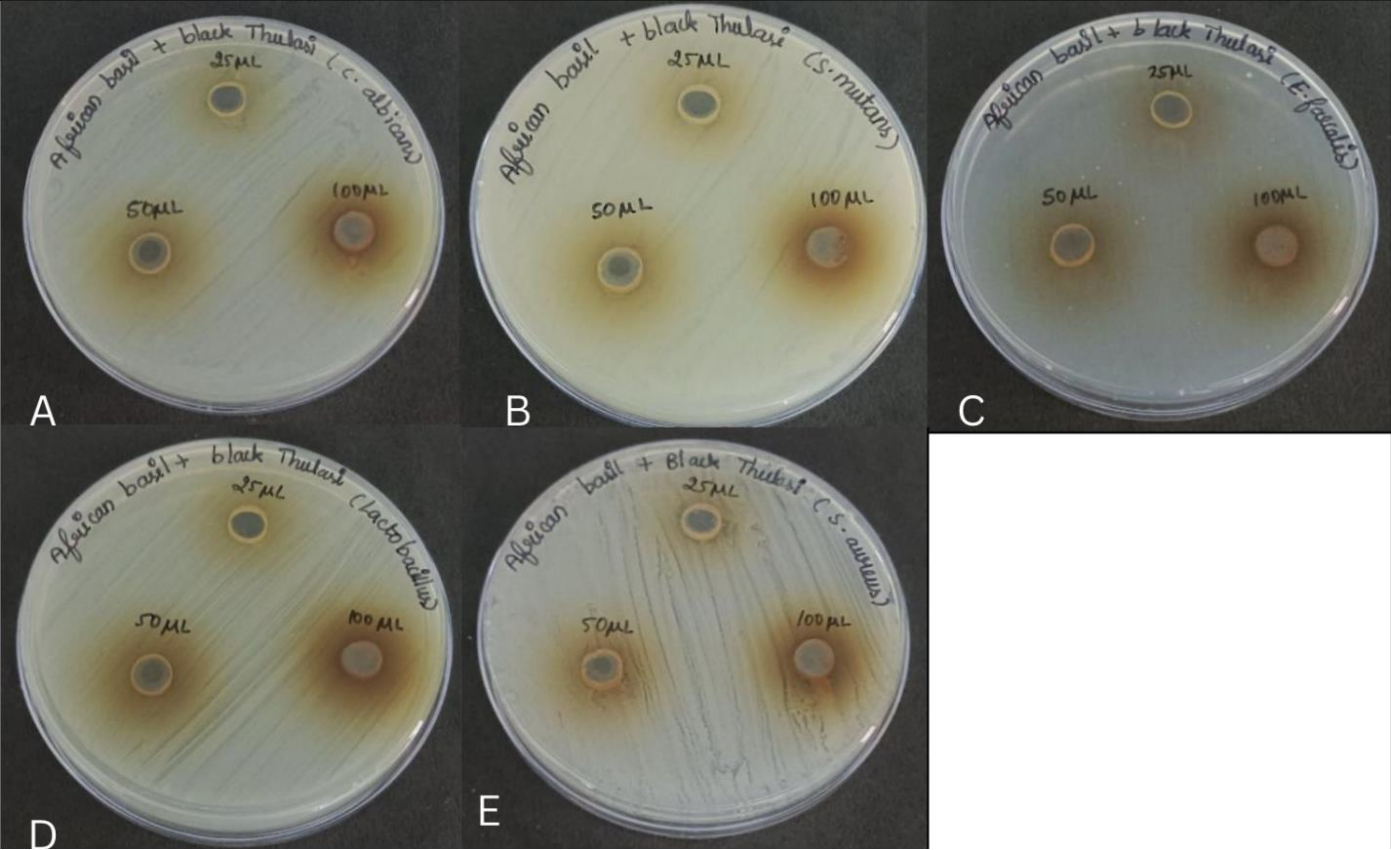
(A-E): Antimicrobial activity of Aftrican bil and Black Tulsi against common oral pathogens at different concentrations using agar well diffusion method.

The antibacterial efficacy of various doses of African Basil and against oral pathogens like *S. mutans,* *Enterococcus faecalis*, *C. albicans*, *Lactobacillus sp,* and *S. aureus* was assessed using the agar well diffusion method. Using a sterile spreader, secondary cultures of microbial suspension were equally distributed on the Muller Hinton agar and the rose Bengal agar plates' surfaces. Through the use of a sterile cork borer and a sterile micropipette, different concentrations of nanoparticles (25, 50, and 100 l) were added to the wells made on the agar plate. The solution's SeNP concentration was 10 mg per 100 ml. After that, the plates were incubated for 24 to 48 hours at 37°C. For *S. mutans,* E. faecalis, Lactobacillus sp., *S. aureus* and Candida albicans. Each plate's ZOI (mm) was measured and contrasted with SeNPs' values. For analysis, every test was carried out triplicated for analysis (Figure 2).

## Assessment of minimum inhibitory concentration:

According to Clinical and Laboratory Standards Institute (CLSI) recommendations, the MIC of the African Basil and Black Tulsi formulation was also measured using the broth microdilution method [16] A stock concentration of 1024 µg mL-1 was used to prepare the samples under examination. A concentration of 512 µg mL-1 was achieved by mixing 500 l of the stock solution with 500 l of MHB medium for bacterial cultures. To obtain 256, 128, 64, 32, 16 and 8 µg mL-1, two-fold serial dilutions were carried out. To each tube of various NPS concentrations, 50 L of microbiological solutions containing 1 106 CFU mL1 were added. Finally, the formulation was developed at doses of 25, 50 and 100 µg mL1.

The control sample (positive control) only contains 100 µl of bacteria in cell culture medium that accurately depicts the growth of the bacteria when NPS is not present, and amoxyrite was chosen as the standard. The final suspension of the bacteria was diluted for each strain and then incubated for 24 hours at 37 °C. The growth of the bacterial strains was assessed via ocular observation after a 24-hour incubation period. The level of NPS required for maintaining the MIC for preventing bacterial growth.

## Cytotoxic effect:

**Figure 3:**
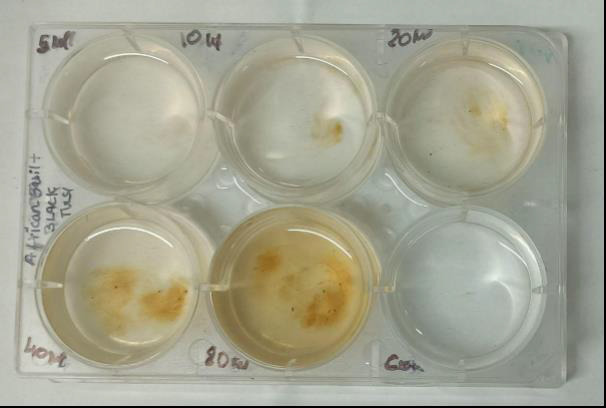
Cytotoxic activity of African basil and Black Tulsi formulation with Nauplii fish at different concentrations

Brine Shrimp Lethality Assay was used to evaluate the cytotoxicity profile of the synthesized SeNPs. Aquatic Remedies, Chennai Pvt. Ltd. provided the brine shrimp eggs. For the purpose of hatching the shrimp eggs, one litre of distilled water was mixed with 36 g of sea salt. A partitioned hatching room with dark/covered and light/exposed portions served as the home for the saltwater. In the chamber's dark side, shrimp eggs were placed; a lamp above the chamber's light side will draw the hatchling shrimps. Ten brine shrimp were placed in test tubes containing 5 ml of synthetic seawater and 5 ml of a nanoparticle solution at various concentrations after the shrimps had been allowed to hatch and develop as nauplii (larva) for two days. The usual control for the test consisted of brine shrimp in 10 cc of synthetic saltwater. The test tubes were left exposed under the light for 24 hours, and the number of shrimps that survived was counted and noted (Figure 3).

## Statistical analysis:

In order to conduct the statistical analysis, SPSS v23.0 (IBM Corp., Armonk, NY, USA) was used. Kolmogorov-Smirnov and Shapiro-Wilk tests were used in the normality test study, which revealed a non-parametric distribution. Data were analyzed utilising mean, standard deviation and percentages to compare absorbance between treatment groups at various time intervals of 530 nm.

## Result

**Figure 4:**
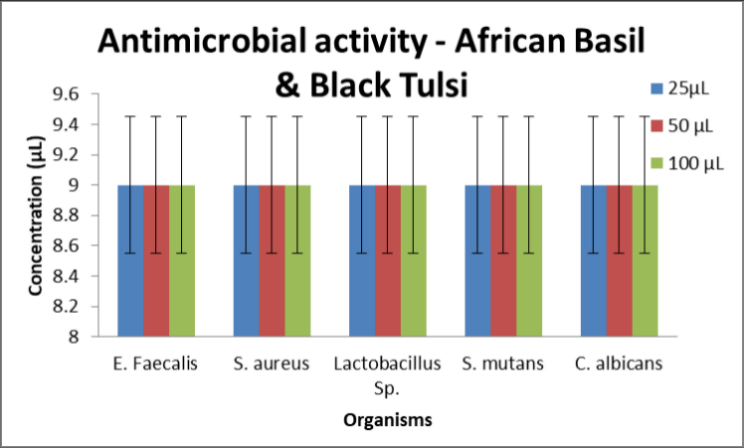
Antimicrobial activity of African basil and Black Tulsi against against common oral pathogens at different concentrations

**Figure 5:**
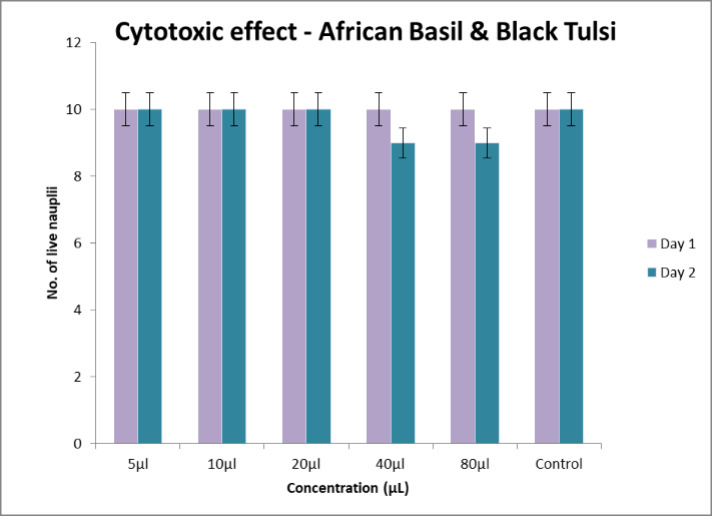
Cytotoxic activity of African basil and Black Tulsi formulation with Nauplii fish at different concentrations

**Figure 6:**
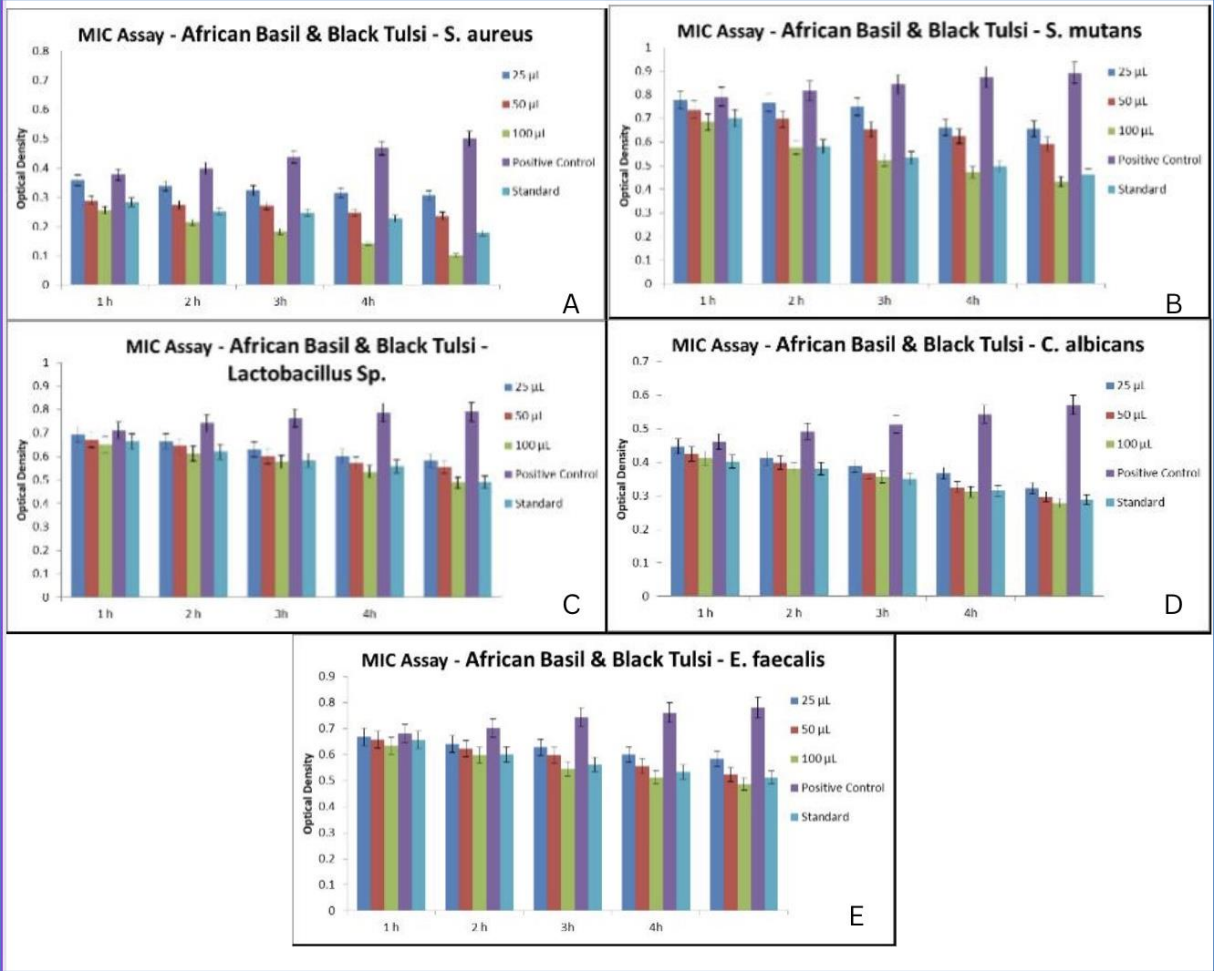
(A-E): Minimum Inhibitory Concentration of African basil and Black tulsi against oral pathogens at different concentrations

Agar well diffusion method was used to determine the antimicrobial activity of different concentrations of African basil and Black Tulsi formulation against strains of *S. mutans,* *S. aureus*, E. faecalis, *C. albicans* and Lactobacillus sp. It was observed that at all concentrations the sample showed antimicrobial activities. Surprisingly, the minimum zone of inhibition (ZOI) was found to be the same with every concentration (Figure 4). Results revealed that on day 1, at all concentrations of the formulation the nauplii were alive. However on day 2, till 20 µl, all nauplii were alive, But on 40 and 80 µl, 90% of nauplii were alive (Figure 5). They are considered low toxicity agents based on Organization for Economic Co-operation and Development guidelines. (Table 1) denotes the MIC value of the formulation which was valued at 100 mg/L at different time intervals. In all the samples, 100 µL concentration formulations had more antimicrobial activity against the oral pathogens than standard and positive control at 5 hr time interval. Number of colonies was more in 25µL concentration and significantly reduced in 100µL. From the graph, it is analysed that the amount of colonies of oral pathogens reduced with increase in concentration of African basil and Black Tulsi (Figure 6).

## Discussion:

Antibiotic drug resistance (AMDR) has developed as a result of the indiscriminate use of antibiotic feed additives in animal husbandry and has been made worse by patients who do not adhere to the recommended antimicrobial dosing regimens[17]. The plant kingdom is the natural resource we have at our disposal for developing new tactics to combat antibiotic resistance. As a result of their secondary metabolites, plant extracts have been found in numerous studies to have effective anti-infective, antioxidant, and anti-inflammatory properties. A number of Ocimum (basil) species have also been shown to possess antibacterial, antifungal, anthelmintic, larvicidal, nematocidal, and gastric cytoprotective antiulcer properties [18]. In this present study, *Ocimum gratissimum* L. and Ocimum tenuiflorum were combined and assessed for antimicrobial activity. Though agar well diffusion method showed no antimicrobial activity, minimal inhibitory concentration of 100µL had better bacteriostatic action than the standard and positive control at almost all time intervals. The formulation was effective against all of the microorganisms evaluated including fungicidal for Candida albicans which was proven with minimal inhibitory concentration assay.

In a similar study, when *Ocimum sanctum* is assessed for MIC, they had 125 mg/ml concentration against Staphylococcus aureus, *E. coli* and *Streptococcus* species [19]. According to Singh *et al.*, O. sanctum oil possesses effective antibacterial properties against B. pumilus, P. aeruginosa, and *S. aureus*. They came to the conclusion that the oil's greater linolenic acid concentration may be a factor in this antibacterial activity. [20] The main components of *O. sanctum*, including volatile oil (Eugenol), linolenic acid, flavonoids, and triterpine, may be responsible for the antibacterial activity of the plant (Ursolic acid). The hydroalcoholic (1:1) extract of *O. sanctum* leaves showed greater zones of growth inhibition against common mastitis pathogens, according to preliminary studies, and this herb's antibacterial potential could be used in in vivo therapeutic trials to demonstrate its usefulness for mastitis prevention [21]. Similarly, *Ocimum gratissimum* at 31 mg/ml revealed least inhibitory zones for Escherichia coli, Staphylococcus aureus, Salmonella spp. Proteus mirabilis and 62.25mg/ml for Pseudomonas aeruginosa and Streptococcus pneumonia. In the same study, *S. aureus* had the highest zone of inhibition [22]. In another study, *Ocimum gratissimum* have a broad spectrum of antibacterial activities. The pathogens *S. aureus* and *E. coli* are associated with nosocomial infections [23,24]. The present study shows not only antibacterial but also anti fungal activity. Similarly *Ocimum gratissimum* has shown antifungal activity against candida species [9,25]. On the other hand, *Ocimum sanctum* exhibited antifungal activity against subcultures of candida albicans [26]. In the present study, the african basil and black tulsi formulation has very less cytotoxicity while assessed with brine shrimp lethality assay. Similarly, when biosynthesized silver nanoparticles (with *O. sanctum*) were assessed for cytotoxicity, they also showed acceptable cytotoxicity [27]. Similarly, *Ocimum gratissimum* in oil formulation showed antioxidant potential, anti-parasite and low cytotoxicity [28,29]. This study proves that the formulation (*O.gratissimum* and *O. sanctum*) synergistically show antibacterial, antifungal and cytotoxic activity. But the major limitation of this study is there was no control while testing the antimicrobial activity. Another limitation is that its a invitro study. The study needs to be more explored in invivo environment.

## Conclusion:

The formulation of *O. gratissimum* and *O. sanctum* are a boon for creating new medications and food preservatives with antibacterial properties. They are effective in battling bacterial and fungi pathogens that are resistant to antibiotics. They are a better alternative to synthetic medications as a secure natural product and are highly suggested for the food, fragrance, and pharmaceutical industries. In the current climate, more attention should be placed on identifying antimicrobial components in plant oils in order to create powerful medications that can counteract the effects of antibiotic-resistant microorganisms. Also, the goal should be to create new blended goods by mixing antibiotics or other chemical compounds with clove basil oil, which exhibits a special synergistic function and has numerous uses in the culinary and pharmaceutical industries.

## Funding:

Nil

## Figures and Tables

**Table 1:** Minimum inhibitory Concentration of of African basil and Black tulsi against oral pathogens at different concentrations

	***1hr***	***2hr***	***3hr***	***4hr***	***5hr***
***S.aureus***
25µL	0.357	0.338	0.323	0.313	0.306
50µL	0.289	0,273	0.27	0.247	0.236
100µL	0.256	0.213	0.182	0.141	0.102
Positivecontrol	0.376	0.398	0.437	0.467	0.499
Standard	0.283	0.251	0.246	0.227	0.178
***S.mutans***
25µL	0.778	0.768	0.75	0.663	0.656
50µL	0.737	0.698	0.654	0.625	0.592
100µL	0.685	0.598	0.525	0.473	0.432
Positivecontrol	0.792	0.817	0.844	0.875	0.893
Standard	0.702	0.582	0.534	0.498	0.462
***Lactobacillussp.***
25µL	0.694	0.665	0.631	0.602	0.583
50µL	0.672	0.645	0.601	0.572	0.555
100µL	0.651	0.613	0.577	0.536	0.489
Positivecontrol	0.712	0.743	0.766	0.787	0.791
Standard	0.665	0.621	0.584	0.559	0.492
***Candidaalbicans***
25µL	0.447	0.412	0.389	0.367	0.323
50µL	0.425	0.398	0.367	0.325	0.297
100µL	0.412	0.379	0.356	0.312	0.278
Positivecontrol	0.461	0.492	0.512	0.543	0.571
Standard	0.402	0.381	0.351	0.315	0.288
***E.faecalis***
25µL	0.669	0.641	0.628	0.601	0.584
50µL	0.658	0.623	0.598	0.556	0.524
100µL	0.634	0.598	0.546	0.512	0.487
Positivecontrol	0.681	0.702	0.744	0.761	0.781
Standard	0.656	0.601	0.562	0.534	0.512
